# Impact of a medical scribe on clinical efficiency and quality in an academic general internal medicine practice

**DOI:** 10.1186/s12913-021-06710-y

**Published:** 2021-07-11

**Authors:** Anastasia Pozdnyakova Piersa, Neda Laiteerapong, Sandra A. Ham, Felipe Fernandez del Castillo, Sachin Shah, Deborah L. Burnet, Wei Wei Lee

**Affiliations:** 1 Pritzker School of Medicine, University of Chicago, Chicago, USA; 2 Department of Medicine, University of Chicago, Chicago, IL USA; 3 University of Chicago Center for Health and the Social Sciences, Chicago, USA

**Keywords:** Scribe, Electronic health records, Primary care, Clinical efficiency, Quality of care

## Abstract

**Background:**

Scribes have been proposed as an intervention to decrease physician electronic health record (EHR) workload and improve clinical quality. We aimed to assess the impact of a scribe on clinical efficiency and quality in an academic internal medicine practice.

**Methods:**

Six faculty physicians worked with one scribe at an urban academic general internal medicine clinic April through June 2017. Patient visits during the 3 months prior to intervention (baseline, *n* = 789), unscribed visits during the intervention (concurrent control, *n* = 605), and scribed visits (*n* = 579) were included in the study**.** Clinical efficiency outcomes included time to close encounter, patient time in clinic, and number of visits per clinic session. Quality outcomes included EHR note quality, rates of medication and immunization review, population of patient instructions, reconciliation of outside information, and completion of preventative health recommendations.

**Results:**

Median time to close encounter (IQR) was lower for scribed visits [0.4 (4.8) days] compared to baseline and unscribed visits [1.2 (5.9) and 2.9 (5.4) days, both *p* < 0.001]. Scribed notes were more likely to have a clear history of present illness (HPI) [OR = 7.30 (2.35–22.7), *p* = 0.001] and sufficient HPI information [OR = 2.21 (1.13–4.35), *p* = 0.02] compared to unscribed notes. Physicians were more likely to review the medication list during scribed vs. baseline visits [OR = 1.70 (1.22–2.35), *p* = 0.002]. No differences were found in the number of visits per clinic session, patient time in clinic, completion of preventative health recommendations, or other outcomes.

**Conclusions:**

Working with a scribe in an academic internal medicine practice was associated with more timely documentation.

**Supplementary Information:**

The online version contains supplementary material available at 10.1186/s12913-021-06710-y.

## Background

In the decade since the Health Information Technology for Economic and Clinical Health (HITECH) Act in 2009, electronic health record (EHR) use has significantly increased in the U.S. [1–3] EHR use has been found to improve some quality and safety measures [4–6]. However, concerns have been raised about increased administrative burdens placed on physicians due to EHR use, and the associated physician burnout [7–13]. Recent studies have found that physicians in outpatient practice spend half their workday on the EHR (e.g., chart review, composition of progress notes, order entry, etc.) and an additional 1–2 h per day outside of office hours on EHR-related work [14–16]. These troubling trends have sparked calls for reductions in EHR-related administrative tasks to enhance practice efficiency and combat physician burnout [17–19].

Scribes are trained personnel who assist physicians with documentation and clinical workflow and are a proposed solution to decrease physician EHR burden [20–23]. A PubMed literature search revealed that scribe use was associated with improvements in clinician satisfaction in emergency departments, and subspecialty and primary care clinic settings [23–29]. In addition, scribes have been found to increase productivity and efficiency in emergency departments and subspecialty clinics [23, 25, 26, 29–34].

Few studies have focused on the impact of scribes on clinical efficiency and quality in primary care. Four previous studies in primary care have found that scribes improved physician productivity, timely chart completion, and EHR note quality [33–36]. However, no study has simultaneously assessed the impact of scribes on clinical efficiency and quality in primary care. We sought to assess the impact of scribes in primary care on clinical efficiency measures (e.g., number of visits per clinic session) and clinical quality (e.g. EHR note quality, rate of immunization and current medication review, and completion of preventative health recommendations) [37].

## Methods

### Setting and participants

We implemented a scribe program at an academic general internal medicine (GIM) clinic at UChicago Medicine between April and June 2017 [38]. Among the faculty physicians, 15 physicians were interested in working with a scribe. From these faculty, six faculty were selected to participate in the program based on their clinic schedules to allow for the scribe to be used full time (Monday-Friday 8 AM-5 PM) during the intervention. Participating faculty had between one and four 4-h clinic sessions per week, and we scheduled the scribe to work with faculty for 25–80% of their total clinic sessions, in order to provide a concurrent control and address issues of secular trends and seasonality in the analysis. In prior scribe studies, providers were required to see more patients when they are provided scribes and their scheduling templates were adjusted accordingly [33, 35]. In our study, while we did not ask physicians to increase the number of patients seen during their scribed clinic sessions, they were permitted to add more patients to their clinic schedule if they desired. Thus, our study design would permit us to see if physicians were willing to add more patients to their schedules when scribes were present without extrinsic pressure.

### Intervention

One full-time medical scribe was hired and trained by a professional scribe agency (PhysAssist Scribes, Inc.). The scribe had 1 year of prior scribing experience and received 40 h of general medical terminology training, as well as 4 h of institution-specific EHR ambulatory training. Before the study, the scribe shadowed each physician for one clinic session [38]. Scribe responsibilities included drafting the clinic note and entering after visit summary instructions. Physicians were responsible for entering orders and reviewing, signing, and closing notes. After the study period, physicians completed a standardized exit interview with a study author (WWL) [38].

### Study design

This program evaluation compared group-level means for clinical efficiency and quality in scribe intervention visits vs. two control conditions (baseline visits during February–March 2017 and concurrent unscribed visits during April–June 2017). Scribed visits were compared with both control conditions (baseline and unscribed) for all measures except for EHR note quality, which was only compared to the baseline group. Two control conditions were included to account for secular trends and potential changes to workflow during the intervention period, which may have impacted outcome measures during the unscribed and scribed visits.

### Outcomes

Data for clinical efficiency and quality were abstracted retrospectively from the EHR. The clinical efficiency outcomes were 1) physician time to close encounter, 2) patient time in clinic, and 3) number of visits per clinic session. Clinical quality measures were assessed by 1) comparing EHR note quality between notes documented during scribed and baseline visits, and 2) comparing rates of medication review, immunization review, populating of patient instructions into the after-visit summary, reconciliation of outside information in the EHR, and completion of preventative health recommendations in scribed vs. baseline and unscribed visits.

Physician time to close encounter was defined as the total difference between the time that the provider closed the encounter in the EHR and the patient’s appointment time; this time included time to review, edit, and sign the note and could be during clinic or at home. Patient time in clinic was defined as the difference between patient check out and check in times.

To assess note quality, clinic notes were systematically abstracted from the EHR for baseline (*n* = 75) and intervention (*n* = 75) groups; a total of 150 charts were expected to find significant differences in note quality [36]. The number of charts reviewed per physician was proportional to their clinical load. For each physician, starting from the last clinic session during the study period and going backwards in time, every other chart was extracted from the EHR until the desired number of charts per physician was obtained. Up to six clinic sessions per physician and time period were needed. Physician identifiers and scribe attestations were removed to assure a blinded chart review.

Quality of EHR notes was assessed using measures from 1) the validated QNOTE tool for assessment of outpatient notes [39], 2) the Physician Documentation Quality Instrument (PDQI) tool (“Internal Consistency” measure) [40], and 3) our internal institutional recommendations (e.g. duplications in medication list and problem list). The final note quality instrument included 11 items with each item scored 0 (no), 1 (partial), or 2 (yes), for a maximal possible score of 22 (Additional file 1). Three authors [AP (medical student), FFC (internal medicine resident), and WWL (general internal medicine faculty physician)] coded 10 progress notes separately using the tool and discussed results to resolve discrepancies. This process was repeated iteratively for 20 progress notes, after which consensus was achieved with a high level of inter-rater reliability (Krippendorff’s alpha = 0.89). Two investigators (AP and FFC) then independently coded the 150 included charts.

The number and proportion of preventative health recommendations completed at the visit was assessed for 12 recommendations: depression screening, prediabetes surveillance, diabetes screening, hepatitis C screening, human immunodeficiency virus (HIV) screening, breast cancer and osteoporosis screening, and shingles, tetanus (Tdap/Td), pneumococcal polysaccharide (PPSV23), pneumococcal conjugate (PCV13) and human papillomavirus vaccine (HPV) vaccine administration [37].

To identify which preventative health items were due on the visit date, preventative health measures completed between 1995 and 2017 were extracted from the EHR. A 90-day window was allowed after each patient encounter in the study period to allow time for completion of the preventative care measure. For each patient, the proportion of completed recommendations compared to recommendations due was calculated for each patient encounter. Patients with more than one visit during the study period were excluded from the preventative care analysis because there would be inadequate time for the 90-day follow-up period. Patients who had no preventative health recommendations due at the visit were also excluded from the preventative care analysis.

### Statistical analysis

For clinical efficiency and quality data, we tested unadjusted group differences between the intervention and each control group using Chi-square tests for dichotomous outcomes, Wilcoxon rank sums for ordinal and non-normally distributed continuous outcomes, and t-tests for normally distributed outcomes. Generalized linear mixed models (GLMMs) were used for adjusted analyses to determine the effect of scribe presence on clinical efficiency or quality controlling for study design, baseline physician percentage, and patient demographics. The outcomes we examined include the physician time to close encounter, number of visits per clinic session, patient time in clinic, EHR note quality, medication review, immunization review, population of patient instructions into the after-visit summary, reconciliation of outside information, and preventative health recommendations. Physician was treated as a random effect in all models.

For time to close encounter, patient visits were nested within physician and modeled using repeated measures to account for meaningful differences in case mix that could affect the time to close the encounter. To determine the differences in the number and proportion of appropriate preventative health recommendations addressed, a multilevel logistic regression was conducted by intervention condition, adjusting for patient’s age, gender, and number of preventative health recommendations due. Count data were modeled using a Poisson model, gamma-distributed outcomes used log-linear models, binary and binomial response data were modeled using logistic regressions, and normally distributed outcomes used linear models. The analyses used alpha = 0.5 to define statistical significance and Stata 15 and SAS 9.4 for computations. This project was approved as a quality improvement project by the University of Chicago and therefore did not require an Institutional Review Board (IRB) approval.

## Results

### Patient demographics

Of the 1973 patient visits included in the analysis, 789 were baseline visits, 605 were unscribed visits during the intervention period, and 579 were scribed visits (Additional file 1). A total of 1493 unique patients were included with a mean of 1.39 (Standard Deviation (SD) 0.69) visits per patient. About half of patients were under 65 years old (50.4%) and over half were women (62.5%) (Additional file 1). A total of 1044 patients were included in the analysis of preventative measures (379 baseline, 332 unscribed, 333 scribed) and 150 EHR notes (75 baseline, 75 scribed) were included in the note quality analysis.

### Clinical efficiency

**Fig. 1:**
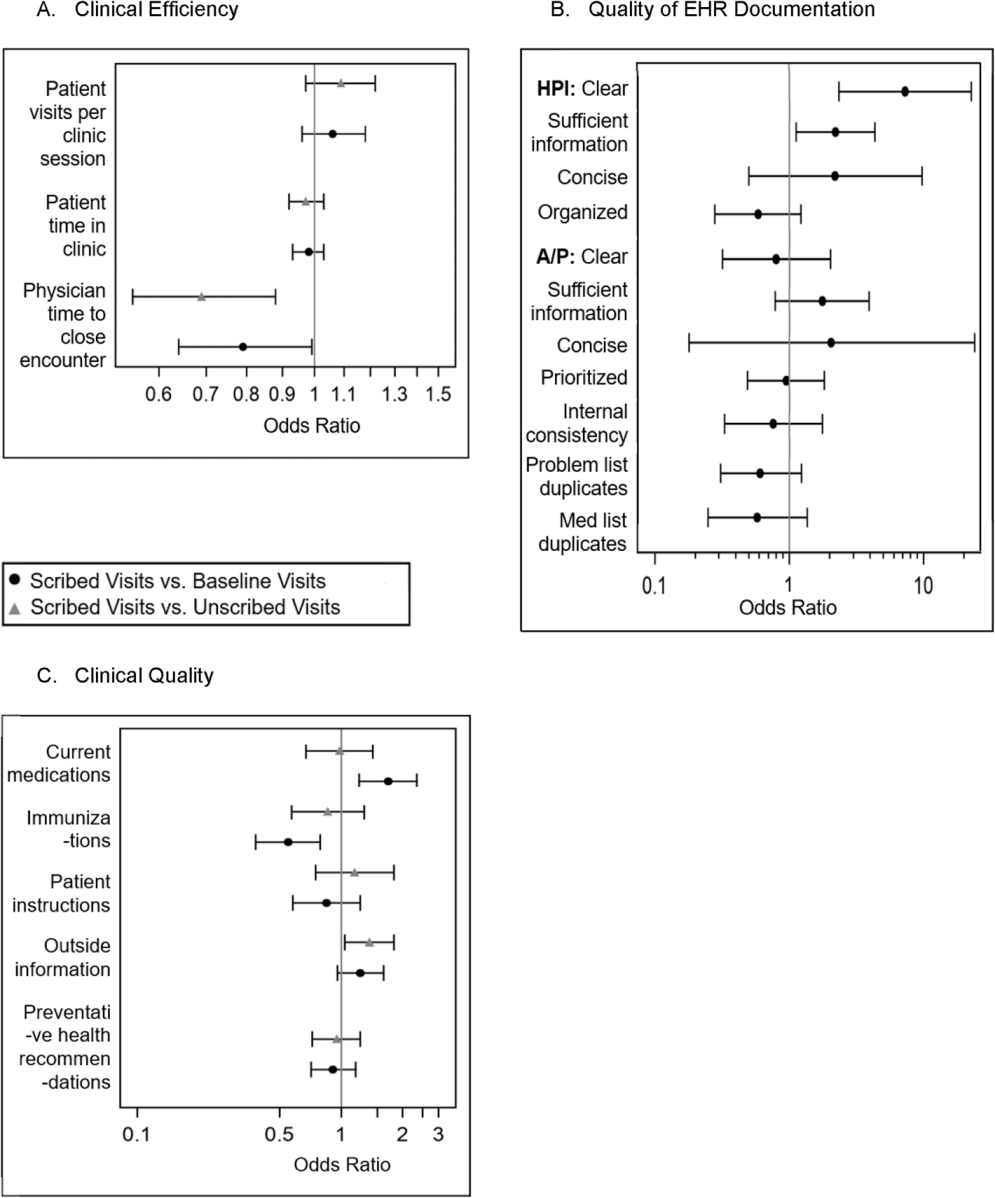
Adjusted Odds Ratios for Models* of Individual Scribe Intervention Outcomes. *Panel **A** Patients per clinic session is adjusted for morning or afternoon clinic and physician mean during baseline control. Visit-level models are adjusted for patient age, gender, and physician mean during baseline control. Odds ratios are estimated from log odds for gamma-distributed outcomes. Panel **B** models are adjusted for physician percentage during baseline control. Panel **C** models adjusted for patient age, gender, and physician percentage during baseline control. All models include physician as a random effect

In unadjusted models, the median (Interquartile Range (IQR)) time to close encounter was shorter for scribed visits [0.4 (4.8) days] than baseline [1.2 (5.9) days] and unscribed visits [2.9 (5.4) days] (both *p* < 0.001). Additionally, there were more patient visits per clinic session during scribed visits [mean (SD), 7.38 (1.94)] than baseline [6.86 (2.11), *p* = 0.08] and unscribed visits [6.58 (2.74), *p* = 0.03] (Table 1). Additional file 1 provides modeling comparisons adjusted for study design characteristics. In adjusted models, the time to close encounter remained shorter during scribed visits compared with baseline and unscribed visits, but there was no longer a difference in the number of patient visits per clinic session (Fig. 1a). Patient time in clinic did not differ in unadjusted or adjusted analyses.

**Table 1:** Clinical efficiency measures for scribed, baseline, and unscribed visits (*n* = 1973)

	Scribed Visits (***n*** = 605)	Baseline Visits (***n*** = 789)	Unscribed Visits (***n*** = 579)	Scribed vs. Baseline Visits ***p***-value	Scribed vs. Unscribed Visits ***p***-value
Patient visits per clinic session, mean (SD)	7.38 (1.94)	6.86 (2.11)	6.58 (2.74)	0.08	0.03
Patient time in clinic, median (IQR) (min)	63.0 (40.0)	63.0 (42.0)	62.0 (40.0)	0.81	0.99
Physician time to close encounter, median (IQR) (days)	0.4 (4.8)	1.2 (5.9)	2.9 (5.4)	< 0.001	< 0.001

### Clinical quality

### Quality of notes

In unadjusted models, the history of present illness (HPI) was rated as more “clear” for scribed visits compared with baseline visits (92.0% vs. 69.3%, *p* < 0.001) (Table 2). In adjusted models, the HPI for scribed notes remained more likely to be rated as “clear” [Odds Ratio (OR) = 7.30, 95% Confidence Interval (CI) 2.35–22.7, *p* = 0.001]. In addition, the HPI for scribed notes was more likely to have “sufficient information” [OR = 2.21, 95% CI 1.13–4.35, *p* = 0.02) (Fig. 1b). There were no differences in the remaining items assessed in the HPI or in the assessment and plan, internal consistency, or number of problem list and medication list duplicates between scribed and baseline notes (Table 2). There was also no difference in the overall note quality score between scribed and baseline notes (average score 15.6/22 for both groups, *p* = 0.92). Of note, in exit interviews, physicians reported that they instructed the scribe to spend less time on the assessment and plan, because they preferred to write this section themselves. Other themes from exit interviews are reported in our previous work [38].

**Table 2:** Unadjusted changes in note quality comparing scribed and baseline visits*

	Baseline Visits (***n*** = 75), n (%)	Scribed Visits (***n*** = 75), n (%)	***p***-value	Odds Ratio (95% CI)^**^	***p***-value
**HPI**					
Clear	52 (69.3)	69 (92.0)	< 0.001	7.30 (2.35–22.70)	0.001
Sufficient information	25 (33.3)	39 (52.0)	0.32	2.21 (1.13–4.35)	0.02
Concise	69 (92.0)	72 (96.0)	0.49	2.20 (0.50–9.76)	0.30
Organized	56 (74.7)	48 (64.0)	0.21	0.59 (0.28–1.22)	0.15
**A/P**					
Clear	65 (86.7)	63 (84.0)	0.82	0.80 (0.32–2.04)	0.64
Sufficient information	14 (18.7)	21 (28.0)	0.25	1.76 (0.79–3.93)	0.17
Concise	73 (97.3)	74 (98.7)	1.00	2.05 (0.18–24.04)	0.56
Prioritized	38 (50.7)	37 (49.3)	1.00	0.95 (0.49–1.83)	0.87
**Internal consistency**	60 (80.0)	57 (76.0)	0.69	0.76 (0.33–1.77)	0.53
**Duplicates in problem list**	30 (40.0)	22 (29.3)	0.23	0.61 (0.31–1.23)	0.17
**Duplicates in medication list**	18 (24.0)	12 (16.0)	0.31	0.58 (0.25–1.36)	0.21
**Total score on Note Quality** Instrument, mean (SD)^***^	15.6 (2.59)	15.6 (2.49)	0.92	0.04 (−0.75–0.83)	0.92

*Abbreviations*: *HPI* history of present illness, *A/P* assessment/plan

* n (%) represents the number and percentage of charts receiving the highest rating (“Yes”) for each category

** Odds ratios represent odds of each note quality characteristic comparing scribed visits to baseline visits accounting for study design

*** Denotes mean total score on note quality instrument, which has a maximum possible score of 22

#### Medication review, immunization review, population of patient instructions, reconciliation of outside information

In unadjusted models, medications were reviewed more frequently during scribed visits compared to baseline visits (88.4% vs. 81.2%, *p* < 0.001) (Table 3). However, immunizations were reviewed less frequently during scribed visits than baseline visits (7.8% vs. 13.8%, *p* < 0.001). In adjusted models (Fig. 1c), current medications (OR = 1.70, 95% CI 1.22–2.35, *p* = 0.002) remained more likely to be reviewed, and immunizations less likely to be reviewed in scribed visits vs. baseline visits (OR = 0.55, 95% CI 0.38–0.79, *p* = 0.002). Additional file 1 includes comparisons between clinical quality measures for scribed, baseline, and unscribed visits adjusted for study design characteristics. Outside information was also more likely to be reconciled for scribed visits compared to baseline in adjusted analyses (OR = 1.37, 95% CI 1.04–1.81, *p* = 0.03) (Fig. 1c). There were no differences in medication or immunization review between scribed and unscribed visits, or population of patient instructions during scribed vs. baseline or unscribed visits.

**Table 3:** Clinical quality measures for scribed, baseline, and unscribed visits

	Scribed (***n*** = 605), n (%)	Baseline (***n*** = 789), n (%)	Unscribed (***n*** = 579), n (%)	Scribed vs. Baseline ***p***-value	Scribed vs. Unscribed ***p***-value
Reviewed medications	535 (88.4)	641 (81.2)	508 (87.7)	< 0.001	0.71
Reviewed immunizations	47 (7.8)	109 (13.8)	57 (9.8)	< 0.001	0.21
Populated patient instructions	52 (8.6)	76 (9.6)	43 (7.4)	0.51	0.46
Reconciled outside information	455 (75.2)	568 (72.0)	407 (70.3)	0.18	0.06

#### Completion of preventative health recommendations

In total, 1044 (70%) patients were included in the preventative care analysis. The most common preventative health recommendations due at visit were depression screening, HIV screening, and shingles vaccination. On average, four recommendations were due per visit for scribed, baseline, and unscribed visits (Additional file 1). The overall mean and proportion of preventative health items addressed within 90 days of the visit was lower at scribed visits [0.38 (SD 0.70); 9.5%] vs. baseline [0.55 (SD 0.84), *p* = 0.05; 14.4%, *p* < 0.001] and unscribed visits [0.56 (SD 0.98), *p* = 0.005; 14.4%, *p* < 0.001) (Additional file 1). The difference was driven primarily by differences in depression screening rates [scribed 17.2% vs. baseline 26.9% (*p* = 0.004) and unscribed 28.8% (*p* = 0.001)]. In adjusted models, scribed visits were no longer different in the overall mean number or proportion of completed preventative care recommendations (Fig. 1c).

## Discussion

In this study, we found that scribed visits were associated with more timely closure of clinic encounters and increased clarity and completeness in HPI documentation at an academic general internal medicine practice. No difference was found in the number of visits per clinic session, patient time in clinic, or completion of preventative health measures in adjusted analyses. While prior scribe studies in primary care required clinicians to see more patients per clinic session or decrease appointment lengths during the intervention period, we did not implement these measures to minimize disruption and assess for impact on existing workflows [33, 35]. To our knowledge, this is the first scribe study using both baseline and concurrent controls to account for secular trends.

The association of scribed visits with decreased time to close encounter is consistent with prior studies [41]. This finding is important for clinical quality since increased lag time between visit date and note closure can result in documentation omissions and inaccuracies, raising patient safety concerns [42, 43]. In addition, when charts are not closed in a timely fashion, billing delays can result in loss of revenue for clinical practices [44]. Thus, employing a scribe may address important quality measures for practices by improving timeliness and integrity of documentation while also optimizing the practice’s revenue cycle management.

The results of our analysis on note quality, reconciliation of information, and preventative health measure completion can be used to identify target areas for improved clinical quality. We found that scribed notes had higher ratings on clarity and completeness for the HPI section and slightly higher rates of medication review, suggesting that working with scribes may be a viable strategy to improve documentation accuracy. However, no difference was found in the quality of the assessment and plan, which was likely related to participating physicians’ self-reported preference for editing this section of the note themselves.

Interestingly, the lower rate of immunization review and no improvement in the completion of preventative health recommendations during scribed visits may have been an unintended consequence of decreased physician time spent in the EHR during scribed encounters. Our finding in the unadjusted analyses of fewer preventative care recommendations addressed during scribed visits was primarily driven by the lower rates of depression screening when the scribe was present. This finding is unsurprising because in 2017, depression screening was expected to be performed by physicians during clinic visits and physicians may have been less aware that depression screening was due since they spent less time in the EHR during scribed visits. Since 2017, the primary responsibility of depression screening at our institution has shifted from physicians to other members of the care team, as is typical in many primary care practices [45].

Our findings on the quality of scribed notes address concerns about the quality of documentation written by scribes or physicians [46]. One prior study found that only 18% of the physician-generated progress note text was manually entered (vs. copy and pasted) by the physician [47], and another study noted that 20% of patients who read their ambulatory note found a mistake in it [48]. As initiatives such as OpenNotes, which promotes note sharing with patients, gain popularity [49, 50], the quality and readability of EHR notes co-authored by scribes may reduce patient confusion and improve patient satisfaction with their notes [33, 41]. Additionally, prior studies have found large physician-physician variability in the content and completion of common clinical documentation domains, which may result in inefficiencies and potential harm to patients due to missed or misinterpreted information [51–54]. Working with scribes may be one strategy to help reduce note variability and improve quality of documentation. Future work should study the impact of enhanced training and providing continuous quality improvement feedback for scribes and physicians to improve clinical quality and documentation [55].

Consistent with prior studies, we found that scribe presence did not impact patient time from check in to check out, which likely reflects the impact of other variables such as clinic staffing ratios and physician time allotted per visit [33, 56]. Importantly, since the scribe was responsible for documentation during clinic visits, it is likely that physicians had increased face-to-face time with patients [33], which may improve doctor-patient relationship, and may lead to patient engagement and improved adherence to care plans [57, 58]. Indeed, while this was not a measured outcome of our study, several participating physicians gave us qualitative feedback that working with the scribe allowed them to more meaningfully engage with patients, increased their facetime with patients, and increased physicians’ satisfaction in their practice [38]. Further, in a prior study of this program, we found that physicians reported spending significantly less time on post-clinic EHR documentation after clinic sessions with a scribe vs. without a scribe [38].

Our study had several limitations. The short duration of the single-site program, small number of physicians participating in the pilot, and employment of a single, well-trained, scribe limit the generalizability of the study. However, our findings are consistent with similar studies in primary care settings finding decreased time to close encounter and no effect on patient time in clinic, which may alleviate some concerns about generalizability [33, 41]. In addition, the GIM clinic at our institution may have similar clinical volume to other large urban academic medical centers. Further, the fact that participating faculty were selected from a group that expressed an interest in working with a scribe may have introduced a selection bias into the study. Additionally, we did not assess how physicians spent their time in the exam room and were not able to objectively assess whether physicians spent more face-to-face time with patients when the scribe was present. Since a relatively small number of EHR notes was reviewed for quality, we also may have missed meaningful note quality differences due to wide confidence intervals around each estimate. Finally, we only accessed an outsourced scribe model, in which training and management of scribes is managed by an outside company, and our results cannot be generalized to in-house scribe models, in which clinical practices expand the roles of team members (e.g. licensed practical nurses or medical assistants) to act as scribes and provide documentation assistance [59–61]. In-house scribe models may be more suitable for certain continuous quality improvement interventions, such as empowering scribes to remind physicians of preventative care gaps.

## Conclusions

Our study found that scribes may improve some aspects of clinical efficiency, quality, and EHR documentation. In addition to these potential benefits, other benefits of scribes that have been previously described including improved physician satisfaction due to decreased administrative burdens of the EHR [7–13]. Importantly, we did not ask clinicians to see more patients when working with a scribe, so our results suggest the impact of a scribe presence on existing clinic workflows. Further research should explore the potential to improve clinical efficiency and quality outcomes in primary care by enhancing workflow training for physicians and scribes and using continuous quality improvement to optimize documentation and address relevant care gaps.

## Supplementary Information

**Additional file 1: Appendix Table 1.** Scoring System for Assessing EHR Note Quality. **Appendix Table 2.** Visit-Level Patient Demographics During Baseline, Unscribed, and Scribed Visits (February–June 2017). **Appendix Table 3.** Comparisons Between Clinical Efficiency Measures for Scribed, Baseline, and Unscribed Visits Adjusting for Study Design (*n* = 1973). **Appendix Table 4.** Comparisons Between Clinical Quality Measures for Scribed, Baseline, and Unscribed Visits Adjusting for Study Design. **Appendix Table 5.** Preventative Health Recommendations Due and Completed within 90 Days for Baseline, Unscribed, and Scribed Visits (*n* = 1044). **Appendix Table 6.** Proportion of Preventative Care Recommendations Completed within 90 Days for Baseline, Unscribed, and Scribed Visits (*n* = 1044).

## Data Availability

The datasets generated and analyzed during the current study are not publicly available to preserve anonymity of deidentified patient data but available from the corresponding author on reasonable request.

## Abbreviations

HITECH: Health Information Technology for Economic and Clinical Health Act of 2009
EHR: Electronic health record
GIM: General internal medicine
PDQI: Physician Documentation Quality Instrument
HIV: Human immunodeficiency virus
Tdap/Td: Tetanus, diphtheria, pertussis vaccine
PPSV 23: Pneumococcal polysaccharide vaccine
PCV13: Pneumococcal conjugate vaccine
HPV: Human papillomavirus
GLMM: Generalized Linear Mixed Models
SD: Standard Deviation
IQR: Interquartile Range
HPI: History of present illness
OR: Odds ratio
CI: Confidence interval
