# Supplementary material for: Impact of a medical scribe on clinical efficiency and quality in an academic general internal medicine practice
Source: BMC Health Serv Res. 2021 Jul 11;21:686. doi: 10.1186/s12913-021-06710-y (PMC8272908; doi:10.1186/s12913-021-06710-y)
Supplement: Supplementary file 1 — Additional file 1: Appendix Table 1. Scoring System for Assessing EHR Note Quality. Appendix Table 2. Visit-Level Patient Demographics During Baseline, Unscribed, and Scribed Visits (February–June 2017). Appendix Table 3. Comparisons Between Clinical Efficiency Measures for Scribed, Baseline, and Unscribed Visits Adjusting for Study Design (n = 1973). Appendix Table 4. Comparisons Between Clinical Quality Measures for Scribed, Baseline, and Unscribed Visits Adjusting for Study Design. Appendix Table 5. Preventative Health Recommendations Due and Completed within 90 Days for Baseline, Unscribed, and Scribed Visits (n = 1044). Appendix Table 6. Proportion of Preventative Care Recommendations Completed within 90 Days for Baseline, Unscribed, and Scribed Visits (n = 1044). [file 12913_2021_6710_MOESM1_ESM.docx]

**Title: Impact of a Medical Scribe on Clinical Efficiency and Quality in an Academic General Internal Medicine Practice**

**Authors:** Anastasia Pozdnyakova Piersa BS^1^, Neda Laiteerapong MD MS^2^, Sandra A. Ham MS^3^, Felipe Fernandez del Castillo MD^2^, Sachin Shah MD**,** MS^2^, Deborah L. Burnet MD MA^2^, Wei Wei Lee MD MPH^2^

^1^Pritzker School of Medicine, ^2^Department of Medicine, University of Chicago, ^3^ University of Chicago Center for Health and the Social Sciences

**Corresponding Author:**

Wei Wei Lee, MD, MPH

Associate Professor

Section of General Internal Medicine

Department of Medicine, University of Chicago

5841 S. Maryland Avenue MC 3051

Chicago IL, 60637

Phone: (773) 834-8435

Fax: (773) 834-7492

Email: [wlee6@uchicago.edu](mailto:wlee6@uchicago.edu)

**Running Title:** Scribe Impact on Clinical Efficiency

References: 63

Tables and Figures: 4

Appendices (in a supplemental file): 4

Text Word Count: 3328

Abstract Word Count: 248

**Key Words:** scribe, electronic health records, primary care, clinical efficiency, quality of care

**Appendix Table 1**. Scoring System for Assessing EHR Note Quality.

| **Measure (Definition)** | **Scoring** | **Source** |
| --- | --- | --- |
| HPI: clear (easy to understand) | Yes=2; Partially=1; No=0 | QNOTE^40^ |
| HPI: sufficient information | Yes=2; Partially=1; No=0 | QNOTE^40^ |
| HPI: concise (focused, not redundant) | Yes=2; Partially=1; No=0 | QNOTE^40^ |
| HPI: organized (properly grouped, chronological) | Yes=2; Partially=1; No=0 | QNOTE^40^ |
| A/P: clear (easy to understand) | Yes=2; Partially=1; No=0 | QNOTE^40^ |
| A/P: sufficient information (pertinent details) | Yes=2; Partially=1; No=0 | QNOTE^40^ |
| A/P: concise (focused, not redundant) | Yes=2; Partially=1; No=0 | QNOTE^40^ |
| A/P: prioritized (in order of importance, includes care plan) | Yes=2; Partially=1; No=0 | QNOTE^40^ |
| Internal consistency (no part of the note contradicts the other part) | Yes=2; No=0 | PDQI^41^ |
| Duplicates in problem list | Yes=2; No=0 |  |
| Duplicates in medication list | Yes=2; No=0 |  |

Abbreviations: HPI, History of Present Illness; A/P, Assessment and Plan.

**Appendix Table 2**. Visit-Level Patient Demographics During Baseline, Unscribed, and Scribed Visits (February-June 2017).

|  | **Overall Visits (n=1,942), n(%)** | **Baseline Visits (n=774), n(%)** | **Unscribed Visits (n=571), n(%)** | **Scribed Visits (n=597), n(%)** | **p-value** |
| --- | --- | --- | --- | --- | --- |
| Age (years), mean (SD) | 62.4 (17.0) | 62.7 (16.7) | 63.6 (17.1) | 61.1 (17.1) | 0.03 |
| 18-39, n (%) | 228 (11.7) | 81 (10.5) | 64 (11.2) | 83 (13.9) | 0.01 |
| 40-64, n (%) | 766 (39.4) | 319 (41.2) | 200 (35.0) | 247 (41.3) |  |
| ≥65, n (%) | 949 (48.8) | 374 (48.3) | 307 (53.8) | 268 (44.8) |  |
| Gender, n (%) |  |  |  |  | 0.78 |
| Female | 1,234 (62.5) | 496 (62.9) | 354 (61.1) | 384 (63.5) |  |
| Male | 708 (35.9) | 278 (35.2) | 217 (37.5) | 213 (35.2) |  |
| Provider, n (%) |  |  |  |  | <0.001 |
| A | 449 (23.1) | 193 (24.9) | 167 (29.3) | 89 (14.9) |  |
| B | 336 (17.3) | 136 (17.6) | 125 (21.9) | 75 (12.6) |  |
| C | 365 (18.8) | 169 (21.8) | 117 (20.5) | 79 (13.2) |  |
| D | 98 (5.1) | 34 (4.4) | 11 (1.9) | 53 (8.9) |  |
| E | 411 (21.2) | 141 (18.2) | 114 (20.0) | 156 (26.1) |  |
| F | 283 (14.6) | 101 (13.1) | 37 (6.5) | 145 (24.3) |  |

**Appendix Table 3.** Comparisons Between Clinical Efficiency Measures for Scribed, Baseline, and Unscribed Visits Adjusting for Study Design (n=1973)

|  | **Unscribed vs. Baseline Visits, OR (95% CI)** | **p-value** | **Scribed vs. Baseline Visits, OR (95% CI)** | **p-value** |
| --- | --- | --- | --- | --- |
| Patient visits per clinic session, mean (SD) | 1.06 (0.96 - 1.18) | 0.26 | 1.09 (0.97 - 1.22) | 0.15 |
| Patient time in clinic, median (IQR) (min) | 0.98 (0.93 - 1.02) | 0.28 | 0.96 (0.92 - 1.01) | 0.13 |
| Physician time to close encounter,  median (IQR) (days) | 0.85 (0.72 - 1.01) | 0.06 | 0.78 (0.65 - 0.94) | 0.01 |

**Appendix Table 4 .** Comparisons Between Clinical Quality Measures for Scribed, Baseline, and Unscribed Visits Adjusting for Study Design

|  | **Unscribed vs. Baseline Visits, OR (95% CI)** | **p-value** | **Scribed vs. Baseline Visits, OR (95% CI)** | **p-value** |
| --- | --- | --- | --- | --- |
| Reviewed  medications | 1.70 (1.22 - 2.35) | 0.002 | 0.98 (0.67 - 1.43) | 0.92 |
| Reviewed  immunizations | 0.55 (0.38 - 0.79) | 0.002 | 0.86 (0.57 - 1.30) | 0.48 |
| Populated patient  instructions | 0.87 (0.59 - 1.27) | 0.47 | 1.18 (0.76 - 1.83) | 0.46 |
| Reconciled outside  information | 1.25 (0.97 - 1.61) | 0.09 | 1.39 (1.06 - 1.84) | 0.02 |

**Appendix Table 5.** Preventative Health Recommendations Due and Completed within 90 Days for Baseline, Unscribed, and Scribed Visits (n=1044).

|  | **Baseline visits (n=366)** | **Unscribed visits**  **(n=319)** | **Scribed visits (n=322)** | **Scribed vs. Baseline**  **p-value** | **Scribed vs. Unscribed**  **p-value** |
| --- | --- | --- | --- | --- | --- |
| Number of  recommendations due at  visit, mean (SD) | 3.81 (1.70) | 3.92 (1.78) | 4.00 (1.74) | 0.17 | 0.48 |
| Number of  recommendations  completed, mean (SD) | 0.55 (0.84) | 0.56 (0.98) | 0.38 (0.70) | 0.005 | 0.05 |
| Percentage of completed  recommendations, mean  (SD) | 14.16 (21.6) | 13.20 (21.5) | 9.81 (18.8) | 0.003 | 0.05 |

**Appendix Table 6**. Proportion of Preventative Care Recommendations Completed within 90 Days for Baseline, Unscribed, and Scribed Visits (n=1044).

|  | **Baseline Visits (n=366)** | | **Unscribed Visits (n=319)** | | **Scribed Visits (n=322)** | | **Scribed vs. Baseline**  **p-value** | **Scribed vs. Unscribed**  **p-value** |
| --- | --- | --- | --- | --- | --- | --- | --- | --- |
|  | **Due n** | **Completed**  **n (%)** | **Due n** | **Completed n (%)** | **Due n** | **Completed n (%)** |  |  |
| Total | 1395 | 201 (14.4) | 1249 | 180 (14.4) | 1289 | 122 (9.5) | <0.001 | <0.001 |
| Depression screening | 320 | 86 (26.9) | 264 | 76 (28.8) | 285 | 49 (17.2) | 0.004 | 0.001 |
| HIV screening | 240 | 16 (6.7) | 217 | 13 (6.0) | 215 | 7 (3.3) | 0.13 | 0.25 |
| Shingles vaccine | 192 | 0 (0.0) | 190 | 0 (0.0) | 172 | 0 (0.0) | -- | -- |
| Tdap/Td vaccine | 167 | 24 (14.4) | 150 | 24 (16.0) | 156 | 15 (9.6) | 0.19 | 0.09 |
| Prediabetes  surveillance | 164 | 36 (22.0) | 144 | 28 (19.4) | 162 | 19 (11.7) | 0.01 | 0.06 |
| Hepatitis C screening | 122 | 14 (11.5) | 90 | 15 (16.7) | 118 | 9 (7.6) | 0.38 | 0.05 |
| PPSV23 vaccine | 76 | 3 (4.0) | 81 | 3 (3.7) | 61 | 1 (1.6) | 0.63 | 0.63 |
| PCV13 vaccine | 49 | 7 (14.3) | 46 | 5 (10.9) | 50 | 8 (16.0) | 1.00 | 0.56 |
| Breast cancer screening | 45 | 7 (15.6) | 42 | 5 (11.9) | 37 | 4 (10.8) | 0.75 | 1.00 |
| Osteoporosis screening | 13 | 2 (15.4) | 9 | 3 (33.3) | 11 | 0 (0.0) | 0.48 | 0.07 |
| Diabetes screening | 6 | 6 (100.0) | 11 | 8 (72.7) | 15 | 9 (60.0) | 0.12 | 0.68 |
| HPV vaccine | 1 | 0 (0.0) | 5 | 0 (0.0) | 7 | 1 (14.3) | 1.00 | 1.00 |

Abbreviations: HIV, Human Immunodeficiency Virus; Tdap/Td, Tetanus, Diphtheria, Pertussis/Tetanus, Diphtheria; PPSV23, Pneumococcal polysaccharide; PCV13, Pneumococcal conjugate; HPV, Human Papilloma Virus.
